# “New” drugs associated with chemsex? 2C-B in sexual context. A case report and review

**DOI:** 10.1192/j.eurpsy.2021.1539

**Published:** 2021-08-13

**Authors:** J. Curto Ramos, H. Dolengevich, M.A. Morillas Romerosa, E. Mateos Pascual

**Affiliations:** 1 La Paz Hospital, Mental Health Unit, Madrid, Spain; 2 Hospital Del Henares, Mental Health Unit, Madrid, Spain; 3 Hospital Juan Ramón Jiménez, Mental Health Unit, huelva, Spain

**Keywords:** chemsex, NPS, MSM

## Abstract

**Introduction:**

The intentional use of drugs before or during sexual intercourse (chemsex), due to its impact on mental health, is a phenomenon of high importance in men who have sex with men.

**Objectives:**

We report the case of a patient with polysubstance acute intoxication, including 2C-B, in order to review the evidence about the mechanisms of action of 2C-B, its efects on sexual pleasure, toxicity, patterns of abuse and somatic and mental health related consequences it may present.

**Methods:**

Case report and narrative review.

**Results:**

We present the case of a patient using 2C-B as a substance in chemsex practice. As the patient presented in our emergency with psychotic symptoms, he was diagnosed with “stimulant acute intoxication” and “acute psychotic symptoms induced by stimulants”. 2C-B increases dopamine (DA) serotonin (5-HT) and norepinephrine (NE) and cause stimulating and hallucinogenic effects.

**Conclusions:**

MSM is a group vulnerable to the problematic use of drugs in a sexual context. Several mental health problems have been associated with chemsex users such as psychotic sypmptoms, suicidal ideation, encephalopaty, delirium. Polysubstance use is common in chemsex practice and it can be difficult to identify the drugs used in states of acute intoxication but psychiatrists must explore the use of differents drugs from the “classic chemsex drugs” (mephedrone, GHB and metanphetamine) including 2C-B and other substances such as cocaine, MDMA, ketamine, and other cathinones different from mephedrone.

